# Spliceosome protein EFTUD2: A potential pathogenetic factor in tumorigenesis and some developmental defects (Review)

**DOI:** 10.3892/mmr.2025.13499

**Published:** 2025-03-19

**Authors:** Ankang Yin, Qiuyu Zhu, Yi Chen, Juan Wang

**Affiliations:** 1 School of Medical Technology and Information Engineering, Zhejiang Chinese Medical University, Hangzhou, Zhejiang 310053, P.R. China; 2 Department of Clinical Laboratory, Tongde Hospital Affiliated to Zhejiang Chinese Medical University (Tongde Hospital of Zhejiang Province), Hangzhou, Zhejiang 310012, P.R. China

**Keywords:** elongation factor tu guanosine-5′-triphosphate binding domain containing 2, RNA splicing, gene mutation, developmental defect, tumorigenesis

## Abstract

The formation of mature mRNA is inseparable from the processing of RNA precursors and splicing by the spliceosome. The spliceosome is a multi-protein complex composed of five small nuclear ribonucleoproteins. Elongation factor Tu GTP binding domain containing 2 (EFTUD2) is a component of spliceosome complex that is involved in the reorganization of the spliceosome complex, thereby promoting the removal of introns from precursor mRNA. Therefore, EFTUD2 can regulate embryonic development and innate immunity by modulating the splicing of various mRNAs. The mutations in EFTUD2 itself also lead to developmental defects and clinical manifestations in mandibulofacial dysostosis, the nervous system, the circulatory system, the digestive system and the reproductive system. Furthermore, the overexpression of EFTUD2 promotes the progression of hepatocellular carcinoma, breast cancer and colorectal cancer. The present review discussed the molecular mechanisms by which EFTUD2 exerts its physiological functions, focusing on EFTUD2 mutations and their corresponding clinical manifestations. It aimed to provide insight for the diagnosis and treatment of EFTUD2-related diseases.

## 1. Introduction

Messenger RNA (mRNA) is a key component in the regulation of gene expression. Before mRNA is formed, pre-messenger RNA (pre-mRNA) is directly transcribed from DNA, but does not have the ability to synthesize proteins. The introns in the pre-mRNA need to be removed for it to become functional, a process carried out by the spliceosome (1). The spliceosome, a multiprotein complex of five small nuclear ribonucleoproteins (snRNPs), recognizes regulatory sequences near intron-exon junctions. By removing introns and splicing exons, it ensures the formation of functional, mature mRNA (2,3). U5-snRNP is one of the core small nuclear ribonucleoproteins of the spliceosome. After U5 recruits U4/U6, they assemble to form the U4/U6-U5 tri-snRNP, which constitutes the precatalytic spliceosome complex (4). The complexity of regulatory elements that control proper splicing makes this process not only crucial to maintain tissue homeostasis, but also highly susceptible to genetic and somatic mutations associated with diseases (5).

Elongation factor Tu GTP binding domain containing 2 (EFTUD2) is an essential protein of U5-snRNP, which is involved in binding GTP and maintaining the normal function of the spliceosome (6). EFTUD2, also known as Snu114, has primary functions in the growth and development of the organism. The EFTUD2 gene is distributed in almost all types of human cells and its mutations can cause biological dysfunctions in various systems. Thus, the present study reviewed current research on EFTUD2 mutations and their clinical implications, focusing particularly on developmental abnormalities, innate immune responses and cancer progression, aiming to offer new insights into the diagnosis and treatment of EFTUD2-related diseases.

## 2. Expression and structure of EFTUD2

**Figure 1.**
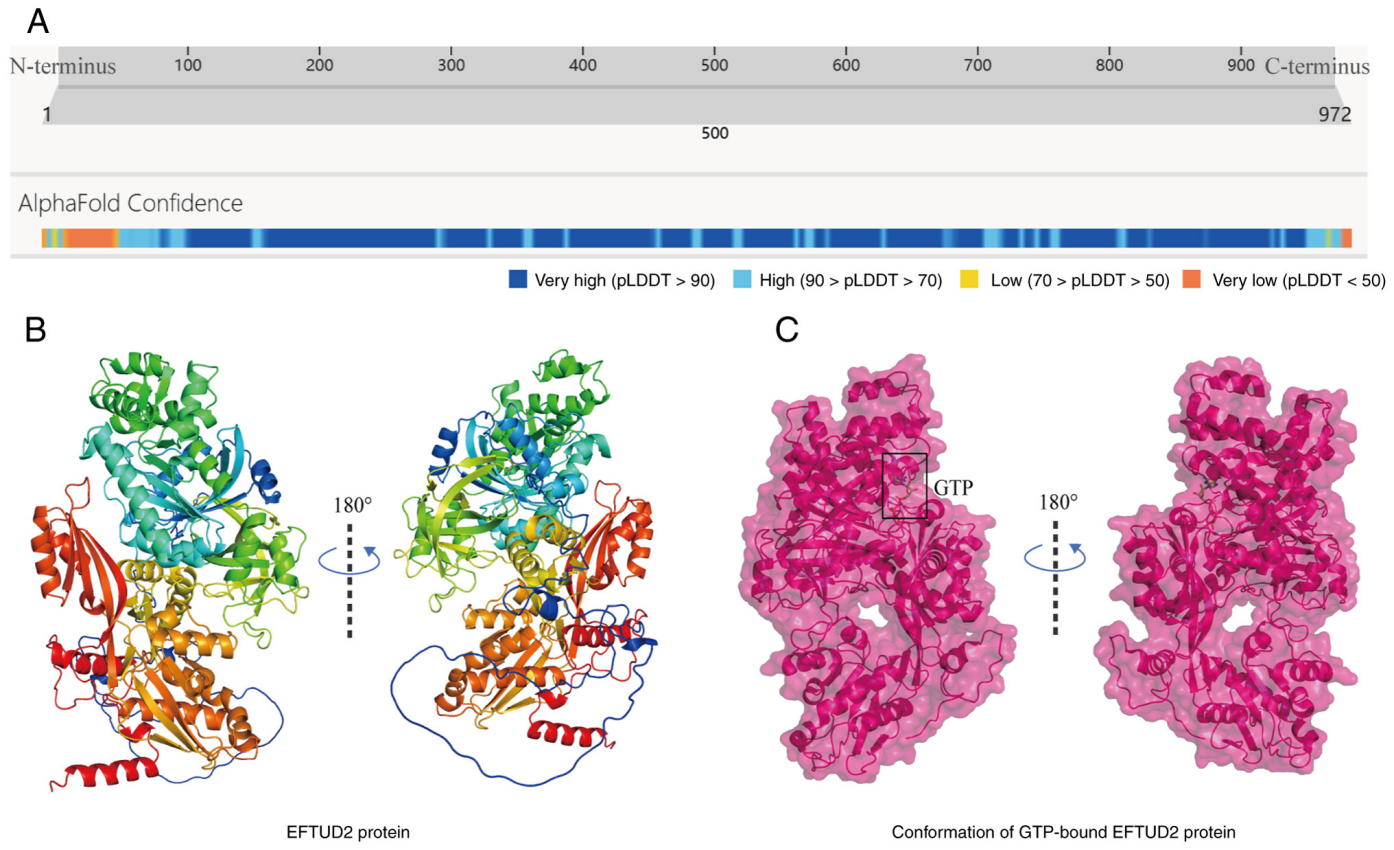
The spatial position and molecular structure of EFTUD2. (A) The spatial confidence analysis of monomeric EFTUD2 (from the AlphaFold Protein Structure Database). (B) The monomeric structure of EFTUD2 (UniProt: Q15029). (C) The molecular structure of EFTUD2 bound to GTP (PDB: 8RC0). The figure was created using Adobe Illustrator (version, 28.3; Adobe Systems, Inc.). EFTUD2, elongation factor Tu GTP binding domain containing 2; GTP, guanosine-5′-triphosphate.

The EFTUD2 gene, located on human chromosome 17q21.31, encodes a ubiquitously expressed 972-amino acid protein with a molecular weight of 109.436 kDa (Fig. 1A) (7). EFTUD2 is also known as MFDGA, U5-116KD and Snu114. The post-translational modifications of EFTUD2 include ubiquitination at Lys352, Lys405, Lys409, Lys581 and Lys790; one glycosylation site according to GlyGen; and one O-linked glycan modification site (8). Mature EFTUD2 contains six functional domains: Elongation Factor G C/N-terminus, Domain III, Domain IV, Elongation Factor Tu Domain 2 and Elongation Factor Tu GTP-binding domain. The monomeric structure of EFTUD2 is shown in Fig. 1B. EFTUD2 shows the highest expression levels in human testis and appendix tissues. Regarding subcellular localization, EFTUD2 is predominantly found in the nucleus, with additional expression observed in the cytoplasm and mitochondria (9). EFTUD2 is also a highly conserved spliceosomal GTPase and an essential component of the spliceosomal complex in cells (6). Plaschka *et al* (10) discovered through cryo-electron microscopy that EFTUD2 binds to GTP but does not appear to hydrolyze GTP to facilitate conformational changes in the spliceosome (Fig. 1C). Therefore, EFTUD2 is more likely to act as a component of a platform that supports precursor mRNA splicing.

## 3. Physiological functions of EFTUD2

### Role of EFTUD2 in splicing

**Figure 2.**
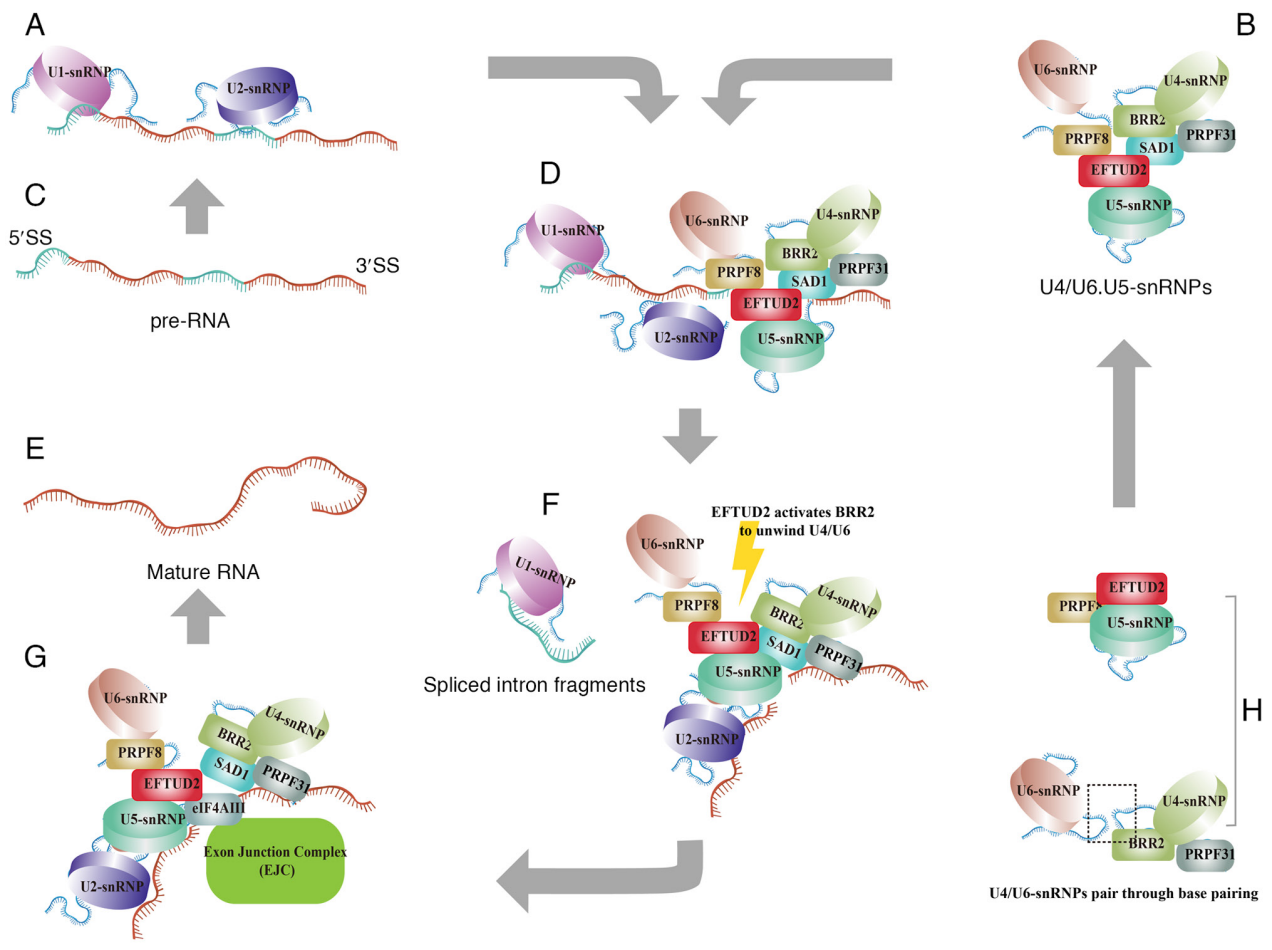
EFTUD2 regulates the removal of introns and the junction of exons. (A) After the production of pre-mRNA, U1 and U2 snRNPs recognize and bind to the 5′ and 3′ ends of the exons, respectively. (B) U4 and U6 snRNPs form a complex through base pairing and then the U5 snRNP, which contains EFTUD2, associates with the U4/U6 complex to form the U4/U6.U5 tri-snRNP complex. (C) Precursor RNA. (D) The U4/U6.U5 tri-snRNP complex subsequently binds to the pre-mRNA already interacted with U1 and U2 snRNPs, initiating the activation of the intron splicing process. (E) Mature RNA. (F) Activation of the intron splicing process. (G) During exon splicing, EFTUD2 interacts with the EJC complex and catalyzes the joining of exons. (H) U4/U6 snRNPs and U5 snRNP. The figure was created using Adobe Illustrator (version, 28.3; Adobe Systems, Inc.). EFTUD2, elongation factor Tu GTP binding domain containing 2; snRNP, small nuclear ribonucleoprotein; EJC, exon junction complex; BRR2, small nuclear ribonucleoprotein U200.

The spliceosome is a large ribonucleoprotein (RNP) complex composed of five snRNPs (U1, U2, U4, U5 and U6) and numerous protein factors, which is assembled *de novo* on each intron (11). Pre-mRNA introns have minimal conserved structural information. The spliceosome recognizes key sequences, such as the 5′ splice site and 3′ splice site and removes introns through splicing to form mature mRNA (3). Specifically, U1 and U2 first bind to the splice sites of the intron (12) (Fig. 2A and C), followed by the recruitment of the U4/U6/U5 tri-snRNP, thereby assembling the precatalytic spliceosome complex (4) (Fig. 2B and H).

EFTUD2 is located in the central region of the tri-snRNP complex, where it interacts with the N-terminal domain of Pre-mRNA Processing Factor 8 (13). EFTUD2 also plays a central role by interacting with Sad1 and UNC84 domain containing 1, which binds to small nuclear ribonucleoprotein U200 (BRR2) and U4/U6-PRPF31. This interaction helps stabilize the association between U5 and U4/U6 snRNPs, ensuring proper spliceosome assembly (14) (Fig. 2D). Then, EFTUD2 regulates the unwinding of U4/U6 by controlling BRR2′s helicase activity, which promotes the spliceosome's transition to an active state (15,16) (Fig. 2F).

The exon junction complex (EJC), which contains eukaryotic translation initiation factor 4A3 (eIF4AIII), is a group of proteins involved in the splicing process, specifically at the exon-exon junctions (17). The RecA1 domain of eIF4AIII directly interacts with EFTUD2, while the EJC recognizes the upstream 5′-exon sequence and binds to EFTUD2 (18) (Fig. 2G). This indicates that EFTUD2 not only serves as a central component of the spliceosome complex, but also actively participates in binding the exon-exon junction (Fig. 2E). Overall, EFTUD2 orchestrates the events required for the correct removal of introns and junctions of exons, thereby influencing gene expression regulation at the post-transcriptional level.

### Role of EFTUD2 in embryonic development

Park *et al* (19) found that EFTUD2 is maternally expressed and remains constant throughout development in *Xenopus* embryos. EFTUD2 is enriched in the anterior neural plate and neural crest formation regions during the neurula stage. While at tailbud stage 29/30, *EFTUD2* transcripts are most abundant in the pharyngeal arches and head. Following *EFTUD2* knockdown, the expression of key neural crest development markers SRY-box transcription factor 9 and SRY-Box transcription factor 2 is reduced in *Xenopus* embryos (19). Thus, the decreased expression of EFTUD2 inhibits the neural crest development of embryos.

A study reported that, compared with heterozygous mutants, *EFTUD2* homozygous mutant embryos exhibit an almost complete absence of the midbrain in neural crest cell mutants by embryonic day 11.5 (20). As embryonic development progresses, neural crest cell-specific EFTUD2 homozygous mutant embryos exhibit severe cranial malformations. By the mid to late embryonic stages, most of these embryos did not survive and the surviving ones displayed exencephaly. Additionally, neural crest cell-specific EFTUD2 homozygous mutant embryos show abnormal trigeminal ganglion formation (20).

However, EFTUD2 heterozygous mutant embryos exhibit developmental delay before organogenesis, but recover by birth (21). Notably, EFTUD2 homozygous mutant embryos are unable to survive post-implantation, a result consistent with the previously findings by Beauchamp *et al* (20,21). Further research revealed that the reduction in EFTUD2 levels led to selective splicing inhibition of double min 2 protein in embryos, resulting in the accumulation of nuclear P53 and increased expression of P53 target genes. Enhanced P53 activity causes abnormal midbrain morphology (20). Whether EFTUD2 affects embryonic development through the regulation of the P53 pathway or other mechanisms remains to be further investigated.

### Role of the EFTUD2 in the innate immune response

**Figure 3.**
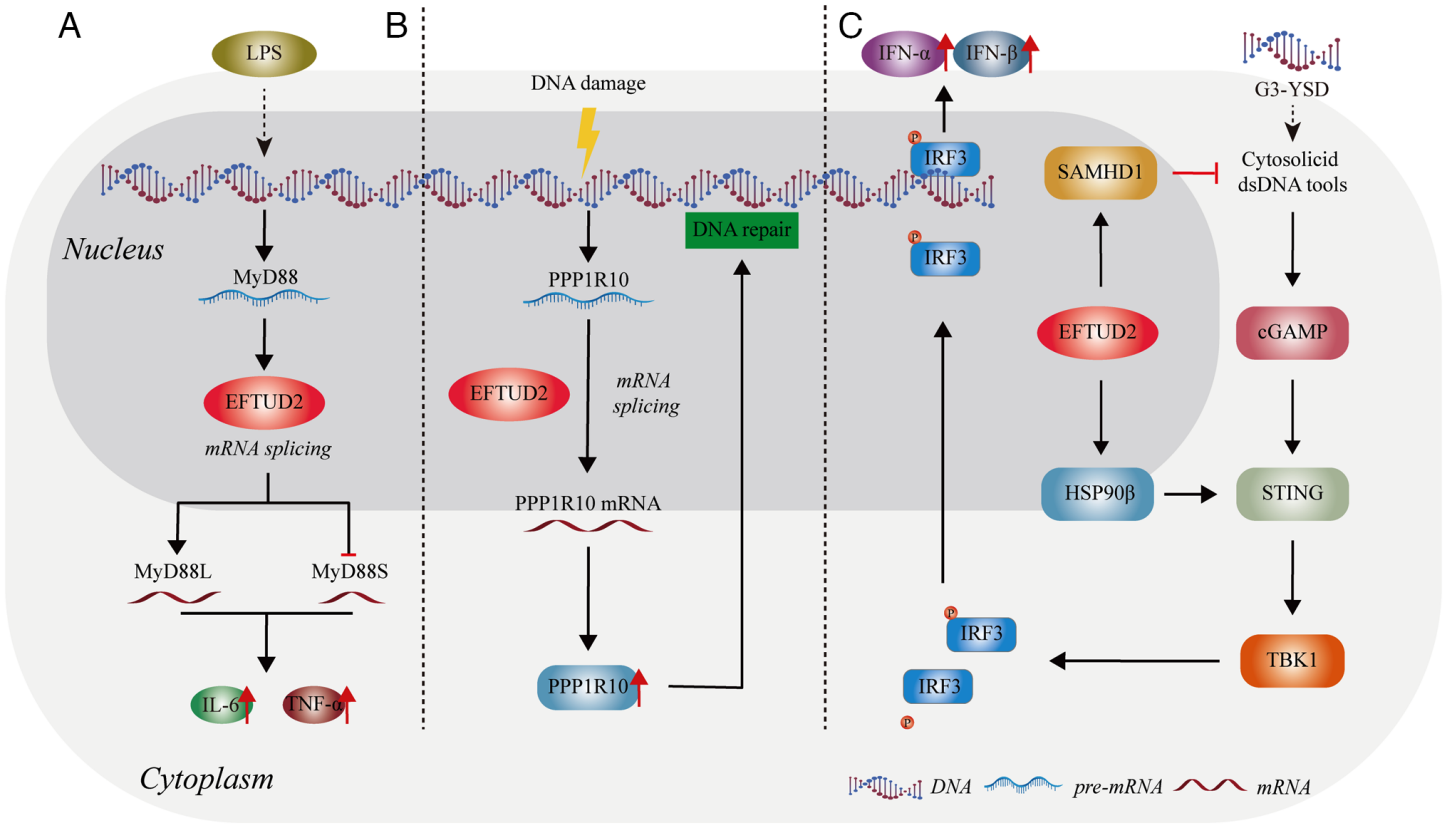
The role of EFTUD2 in the innate immune response. (A) EFTUD2 modulates the activation of the innate immune response by regulating the proportions of MyD88 splice variants. (B) EFTUD2 promotes DNA damage repair by regulating the mRNA splicing of *PPP1R10*, thereby inhibiting inflammatory damage. (C) EFTUD2 is involved in activating the cGAS-STING pathway. The figure was created using Adobe Illustrator (version, 28.3; Adobe Systems, Inc.). EFTUD2, elongation factor Tu GTP binding domain containing 2; MyD88, myeloid differentiation primary response 88; LPS, lipopolysaccharide; IFN, interferon; IRF, interferon regulatory factor; SAMHD1, sterile alpha motif and histidine-aspartate domain-containing protein 1; cGAS, cyclic GMP-AMP synthase; STING, stimulator of interferon response cGAMP interactor; TBK1, tank binding kinase 1.

The innate immune response is the body's first line of defense, preventing infections and targeting invading pathogens (22). Multiple studies have shown that EFTUD2 plays a role in regulating innate immunity (23–25) (Fig. 3).

### Enhancement of immune effects on macrophages

Macrophages are important players in innate immunity, recognizing and effectively responding to invading pathogens, thereby providing an early defense against external attacks (26). De Arras *et al* found that after lipopolysaccharide (LPS) stimulation, EFTUD2 regulates macrophage activation by splicing myeloid differentiation primary response gene 88 (MyD88) pre-mRNA into two forms: *MyD88L* and *MyD88S* (23) (Fig. 3A). MyD88 is an adaptor protein that functions downstream of most Toll-like receptors (TLRs) (27). The full-length MyD88L encodes a critical signaling adaptor protein in multiple TLR response pathways. By contrast, the shorter spliced form, *MyD88S*, which lacks one exon, encodes an in-frame protein that acts as a negative regulator of TLR signaling, preventing downstream signal activation (28). When EFTUD2 is inhibited, a marked increase in the inhibitory spliced form *MyD88S* is observed, along with a concomitant reduction in the production of cytokines interleukin 6 (IL-6) and tumor necrosis factor α (TNFα) (23). Therefore, EFTUD2 can promote the production of IL-6 and TNFα by macrophages by reducing the proportion of *MyD88S* mRNA (Fig. 3A).

White *et al* (24) found that EFTUD2 boosts the production of protein phosphatase 1 regulatory subunit 10 (PPP1R10) in human macrophages by altering its mRNA splicing. The increase in PPP1R10 protein levels of allows the repair of DNA damage in human macrophages, potentially preventing immune damage triggered by cytoplasmic DNA sensors (24) (Fig. 3B). Current research shows that EFTUD2 primarily regulates immune factors through its splicing function. However, the exact mechanisms remain unclear and requires further investigation.

### Regulation of the cyclic GMP-AMP synthase (cGAS)-stimulator of interferon response cGAMP interactor (STING) pathway

The cyclic cGAS-STING pathway is one of the crucial innate immunity pathways. As a highly conserved innate immune signaling mechanism in mammals, activation of the cGAS-STING pathway is characterized by complex transcriptomic changes (29). Sun *et al* (25) reveal that EFTUD2 is predicted to be highly relevant to the cytosolic DNA sensing pathway and shows a high expression correlation with cGAS and STING. Upon overexpression of *EFTUD2*, the number of induced and repressed genes following cGAS-STING activation markedly increases and decreases, respectively. This indicates that EFTUD2 plays a regulatory role in the transcriptomic changes mediated by cGAS-STING pathway activation.

Further research found that overexpression of *EFTUD2* led to the upregulation of heat shock protein 90 β (HSP90β) and sterile alpha motif and histidine-aspartate domain-containing protein 1 (SAMHD1) (25). SAMHD1 is involved in regulating the availability of intracellular nucleic acids and participates in the formation of cGAMP (30). The chaperone protein HSP90β, as a novel STING-interacting protein, modulates STING to promote the activation of tank binding kinase 1 (TBK1) through the aforementioned pathway to phosphorylate interferon regulatory factor 3 (IRF3), facilitating the release of interferon (IFN)α/β (31) (Fig. 3C). Overall, EFTUD2 might control the cGAS-STING pathway to regulate innate immunity.

## 4. EFTUD2 mutations

**Figure 4.**
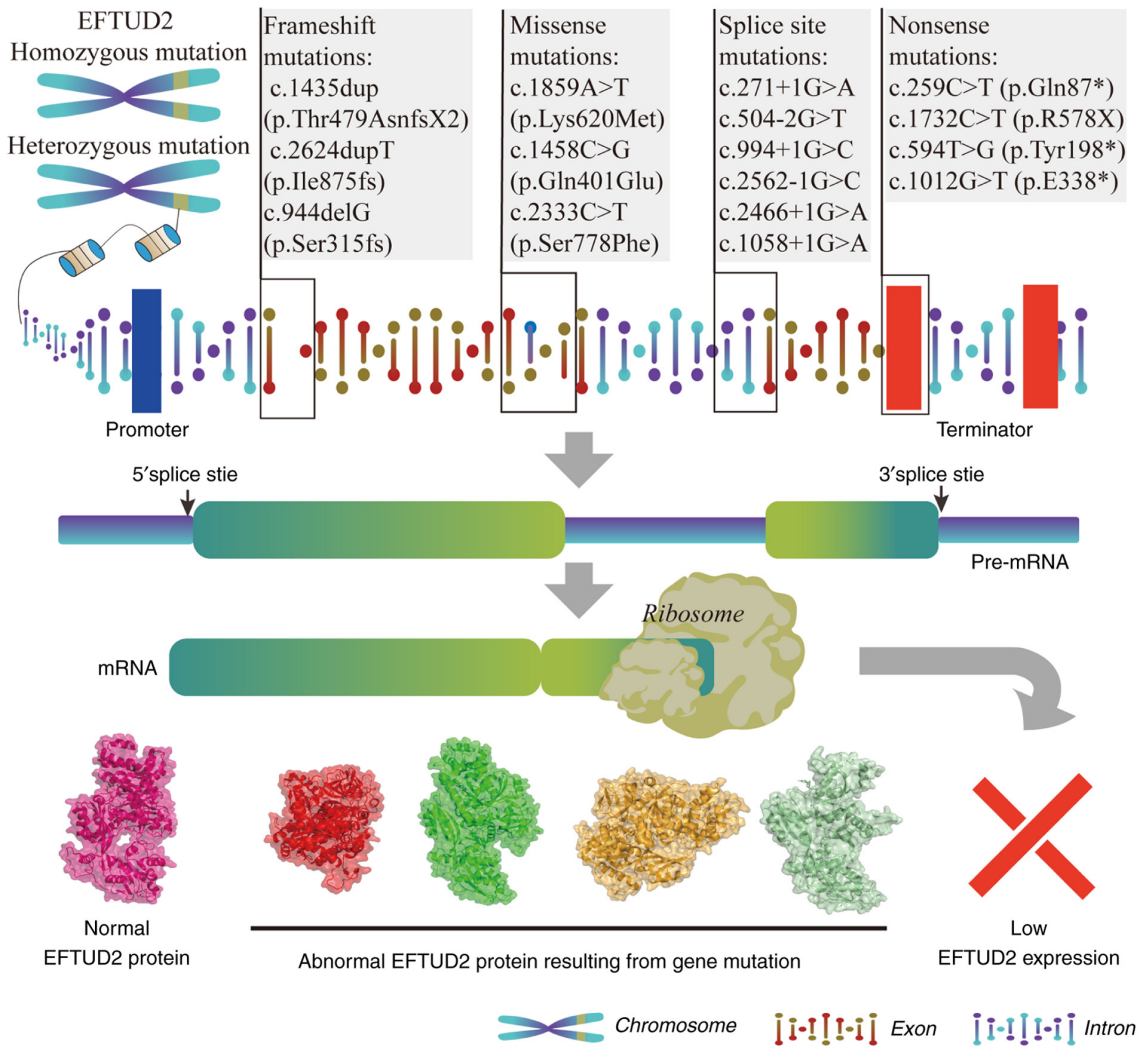
Flowchart illustrating the process from EFTUD2 genomic mutations to abnormal protein expression. The figure was created using Adobe Illustrator (version, 28.3; Adobe Systems, Inc.). EFTUD2, Elongation Factor Tu GTP Binding Domain Containing 2.

Clinical studies have extensively reported *EFTUD2* mutations and their manifestations in humans. According to previous studies, *EFTUD2* mutations primarily cause developmental defects (32–35) (Table I). The process from *EFTUD2* genomic mutations to abnormal protein expression is illustrated in Fig. 4. The main types of *EFTUD2* mutations include frameshift mutations, missense mutations, splice site mutations and nonsense mutations. The clinical symptoms associated with different mutations are shown in Table II.

### Frameshift mutations

Frameshift mutations are caused by insertions or deletions. These mutations disrupt the reading frame of the gene. As a result, they frequently produce premature stop codons, leading to the creation of truncated, non-functional proteins. Sarkar *et al* (32) identified the c.933dupC (p.S312fs) mutation in patients with developmental defects, this mutation is categorized as an insertion/frameshift mutation. It occurs because of the duplication of a nucleotide at position 933, resulting in a shift in the reading frame. Smigiel *et al* (33) also identified the c.1435dup (p.Thr479AsnfsX2) mutation in patients with developmental defects. Similarly, the deletion mutation c.2698_2701del, found in a patient with ventriculomegaly, leads to a frameshift and premature stop codon (34). Another mutation, c.2624dupT (p.Ile875fs), was linked to Guion-Almeida type mandibulofacial dysostosis with microcephaly (MFDM), resulting in a truncated EFTUD2 protein (35). Additionally, a heterozygous c.944delG (p.Ser315fs) frameshift mutation was reported in a male infant with total anomalous pulmonary venous drainage (TAPVD) and his mother (36). A novel frameshift mutation, c.2314del (p.Gln772ArgfsTer21), was also detected in a patient with esophageal atresia/tracheoesophageal fistula (EA/TEF) (37). Khattar and Suhrie (38) detected *EFTUD2* mutations in two patients with EA/TEF, respectively NM_004247.3: c.969del and NM_001258353: c.969del, which are the same mutation occurring at the same position in different *EFTUD2* transcripts. The occurrence of similar mutations in *EFTUD2* across different diseases suggests that frameshift mutations in *EFTUD2* might have a widespread impact on the pathogenesis of these conditions. Further research into the molecular mechanisms is needed to determine whether there are underlying connections.

### Missense mutations

Missense mutations cause a single nucleotide change, replacing one amino acid with another, with varying effects on protein function. Bukowska-Olech *et al* (39) reported two missense mutations in patients with facial dysostoses: c.491A>G (p.Asp164Gly) and c.779T>A (p.Ile260Asn). These two mutations are both classified as missense mutations, leading to the substitution of aspartic acid (Asp) with glycine (Gly) and isoleucine (Ile) with asparagine (Asn), respectively, further resulting in the structural or functional alteration of EFTUD2. In patients with Guion-Almeida type MFDM, the missense mutations c.1859A>T (p.Lys620Met) (33) and c.784C>T (p.Arg262Trp) (40) were identified, which altered the protein structure. Additionally, Lacour *et al* (41) discovered a missense mutation, c.2333C>A (p.Pro778His), in exon 23 of *EFTUD2* in a patient with MFDM. Luquetti *et al* (42) also identified two *de novo* variants associated with MFDM: c.2637G>A (p.Glu794Lys) and c.1458C>G (p.Gln401Glu). From the aforementioned, it is evident that missense mutations in *EFTUD2* typically lead to abnormal development of the human mandible. Whether this mutation will cause other defects remains to be further investigated.

### Splice site mutations

Splice site mutations occur at exon-intron boundaries, disrupting normal RNA splicing and potentially leading to exon skipping or intron retention. In a child with MFDM, Kim *et al* (43) identified a novel splice donor site variant, c.271+1G>A in *EFTUD2*. Minigene assays demonstrated that this variant led to the erroneous integration of a 118 bp fragment from Intervening Sequence 3 (IVS3) of *EFTUD2* in the c.271+1G>A variant clones. This integration produced a truncated EFTUD2 protein, reducing its length by 11.7%. Lacour *et al* (41) identified a novel *de novo* splice site mutation, c.2466+1G>A (IVS24+1G>A), at the splice donor site of *EFTUD2* intron 24. Luquetti *et al* (42) found the *EFTUD2* splice site mutation c.504-2G>T at the acceptor site near exon 504, while Voigt *et al* (44) reported a c.994+1G>C mutation in patients with oculo-auriculo-vertebral spectrum (OAVS). In a patient with generalized seizures, the c.2562-1G>C mutation was identified at the splice acceptor site. Similarly, a splice site mutation in intron 4 of *EFTUD2*, c.351-1G>A (p.Asp117Glufs*8), was detected in a patient with Oto-facial syndrome (44). Another splice site mutation, c.1058+1G>A, was detected in a patient with EA/TEF (38). Splice site mutations in *EFTUD2* are associated with facial developmental defects such as MFDM, OAVS and Oto-facial syndrome. Compared with missense mutations, splice site mutations of *EFTUD2* are more likely to result in a broader range of craniofacial developmental abnormalities.

### Nonsense mutations

Nonsense mutations introduce premature stop codons, truncating the protein and typically resulting in non-functional proteins. The c.259C>T (p.Gln87*) mutation of *EFTUD2* was detected in a patient with mandibulofacial dysostosis (MFD) (45), while another patient with MFDM carried the c.1732C>T (p.R578X) mutation (32), both of which caused premature termination of EFTUD2 protein synthesis. Additionally, Voigt *et al* (44) identified a novel heterozygous mutation, c.594T>G (p.Tyr198*), in a patient with Nager syndrome. A *de novo* nonsense mutation, c.1012G>T (p.E338*), in exon 12 of *EFTUD2* was discovered through whole-genome sequencing in a couple with recurrent miscarriages. This mutation produces a truncated EFTUD2 protein, missing 634 amino acids. Zebrafish models confirmed that this mutation causes EFTUD2 loss of function, affecting hindbrain development and heart formation (46). Notably, besides causing MFDM, nonsense mutations in *EFTUD2* can also lead to miscarriages. Further research is needed to determine whether these miscarriages are related to fetal defects caused by *EFTUD2* mutations.

In summary, the mechanisms of *EFTUD2* mutations have deepened our understanding of the gene; however, to intervene in *EFTUD2* mutations or compensate for the subsequent outcomes caused by *EFTUD2* mutations represents a significant research challenge for the future.

## 5. Role of EFTUD2 in tumors

EFTUD2, as an important component of the spliceosome, is involved in pro-mRNA pruning and splicing, thus its altered expression inevitably plays a crucial role in tumorigenesis and development. Studies found that the expression level of EFTUD2 was markedly increased in tumor tissues (47,48). An elevated level of EFTUD2 is also associated markedly with the prognosis of a variety of tumors and has the potential to be used as a tumor independent prognostic biomarker (Table III).

### Hepatocellular carcinoma (HCC)

**Figure 5.**
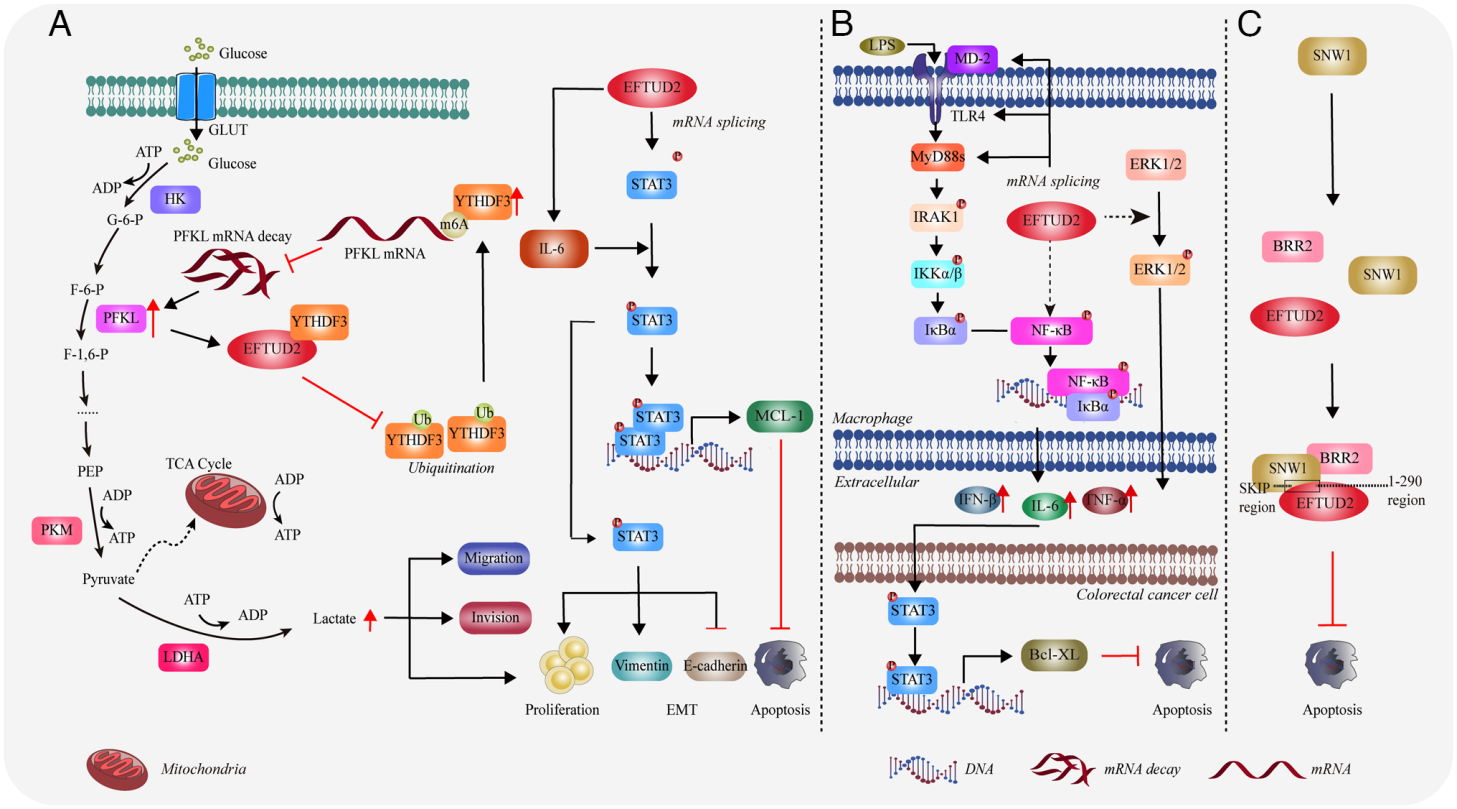
EFTUD2 promotes the development and progression of various tumors. (A) The mechanism by which EFTUD2 promotes the development of hepatocellular carcinoma. (B) The mechanism by which EFTUD2 promotes the development of colorectal cancer through the regulation of macrophage inflammatory response. (C) The role of EFTUD2 in the progression of breast cancer. The figure was created using Adobe Illustrator (version, 28.3; Adobe Systems, Inc.). EFTUD2, elongation factor Tu GTP binding domain containing 2; MyD88, myeloid differentiation primary response 88; GLUT, glucose transporter; ADP, adenosine diphosphate; HK, Hexokinase; PFKL, phosphofructokinase L; YTHDF3, YTH domain family protein 3; LDHA, lactate dehydrogenase A; IL, interleukin; STAT3, signal transducer and activator of transcription 3; IRAK1, interleukin 1 receptor associated kinase 1; MCL-1, myeloid cell leukemia-1; ERK, extracellular signal-regulated kinase; TLR, Toll-like receptor; SNW1, SNW domain containing 1; BRR2, small nuclear ribonucleoprotein U200; NF-κB, nuclear factor kappa B; TNF, tumor necrosis factor.

EFTUD2 is closely associated with the progression of HCC. Studies have reported that EFTUD2 is upregulated in HCC tissues and HCC patients with high levels of EFTUD2 have shorter overall and recurrence-free survival (47,48). Knockdown of *EFTUD2* markedly inhibits HCC cell viability and cell cycle progression, promotes apoptosis and suppresses metastasis. When the expression level of EFTUD2 is reduced, it arrests the cell cycle of liver cancer cells and hinders the transition from the G_1_ to S phase (47,48). Zhou *et al* (49) discovered that EFTUD2 binds to YTH domain family protein 3 (YTHDF3), thereby inhibiting YTHDF3′s ubiquitination. This inhibition leads to an increase in YTHDF3 levels. In turn, the elevated YTHDF3 can suppress the degradation of phosphofructokinase (PFKL) mRNA via m6A modification, thus maintaining *PFKL* mRNA levels. Through this mechanism, EFTUD2 enhances tumor glycolysis in HCC, which promotes the proliferation, migration and invasion of HCC cells (49) (Fig. 5A). Further research showed that EFTUD2 enhances the expression of signal transducer and activator of transcription 3 (STAT3) and cytokine IL-6. IL-6 participates in STAT3 protein phosphorylation, thereby promoting the expression of Myeloid cell leukemia 1 (MCL-1) and vimentin, ultimately leading to EMT and the inhibition of apoptosis (47,50) (Fig. 5A). Zhou *et al* (51) found that EFTUD2 correlates positively with levels of B cells, CD4+ T cells, CD8+ T cells, neutrophils, macrophages and dendritic cells in the tumor microenvironment. This suggests that EFTUD2 is involved in regulating immune infiltration in HCC by promoting the formation of a tumor-favorable immune microenvironment. Thus, EFTUD2 is considered to be a potential therapeutic target for liver cancer (48,51).

### Colorectal cancer

Lv *et al* (52) discovered that EFTUD2 protein levels are abnormally increased in colorectal cancer and reduced EFTUD2 levels leads to a marked decrease in both the number and size of colorectal tumors, but an increase in low-grade dysplasia. The binding of TLR ligands, such as LPS produced by gut microbiota, activates the Toll-like receptor (TLR4)/nuclear factor κB (NF-κB) inflammatory signaling pathways, which are critical factors in the development of colorectal cancer (52,53). EFTUD2 can enhance this pathway by regulating the splicing of mRNAs for components and kinases, including membrane-bound TLR4, full-length myeloid differentiation protein-2 (MD-2) and MyD88L, thereby activating macrophages (52). These activated macrophages then produce and release pro-tumor cytokines such as IL-6, IFN-β and TNF-α (52,54–56) (Fig. 5B). Additionally, EFTUD2 also promotes the activation of the mitogen activated protein kinase (MAPK)/extracellular regulated kinase pathway in macrophages and mouse colon tissues, leading to an increased release of factors such as IL-6 and IFN-β (52) (Fig. 5B). These cytokines promote the proliferation of intestinal epithelial cells by activating STAT3 and its downstream target gene Bcl-XL (52) (Fig. 5B). Bcl-2-like protein 1 3 (Bcl-XL) is an anti-apoptotic protein that plays a critical role in mediating the survival of colorectal epithelial cells (57). Therefore, EFTUD2 deficiency can induce apoptosis in colon cancer cells by inhibiting the secretion of cytokines through the suppression of the TLR/NF-κB signaling pathway and by suppressing the expression of BCL-XL.

Several studies have explored tumor-related inflammatory marker characteristics, including those of colorectal cancer, through blood leukocyte levels (58–60). Whether EFTUD2 contributes to colorectal cancer progression by enhancing this inflammatory response remains to be investigated. Additionally, as aforementioned in sections ‘Enhancement of immune effects on macrophages’ and ‘Regulation of the cGAS-STING pathway’, studies have reported that EFTUD2 is involved in regulating immune responses, such as those related to macrophages (23) and the cGAS-STING pathway (25). While macrophages, as immune cells, have been highlighted in the development of colorectal cancer, the role of other immune cells remains unclear. Therefore, further investigation into the inflammatory characteristics of EFTUD2 in colorectal cancer progression is crucial for improving treatment and prognosis.

### Breast cancer

A study reported that EFTUD2 expression is markedly elevated in breast cancer cells (61). Through immunoprecipitation analysis, researchers found that the 1–260 region of EFTUD2 interacts with SNW domain containing 1 (SNW1) 174–335 region (SKIP domain). SNW1 is a highly conserved protein that functions as a splicing factor in RNA transcription and splicing and its deficiency can lead to splicing defects (62).

Additionally, EFTUD2 connects the C-terminus of BRR2 to the SKIP domain of SNW1 through its N-terminus, thereby forming a complex protein structure (Fig. 5C). Researchers expressed *EFTUD2* and *SNW1* deletion mutants in breast cancer cells to disrupt the EFTUD2-SNW1-BRR2 complex. They found that >50% of cells with these mutants experienced apoptosis (61) (Fig. 5C). Thus, the interaction between EFTUD2 and SNW1 is crucial for the survival of breast cancer cells, because its disruption will lead to increased apoptosis.

### Other types of cancers

Investigators found that EFTUD2 had the highest risk score in the development of a prognostic risk score model for bladder cancer, indicating its significant predictive value for patient outcomes (63). In addition, a clinical study has shown that high expression of EFTUD2 in endometrial cancer is predictive of poor prognosis (64). In multivariate Cox regression analysis, EFTUD2 was identified as an independent marker for progression-free survival in endometrial cancer and could serve as a negative prognostic indicator for patients (64). Research on EFTUD2 in bladder and endometrial cancer is currently limited to data analysis. Further cellular and *in vivo* experiments are required to explore the mechanisms by which EFTUD2 influences the development and progression of this tumor.

In summary, the evidence linking EFTUD2 to poor prognosis in cancers suggests that it could serve as a valuable biomarker to predict patient outcomes. Furthermore, the role of EFTUD2 in shaping the tumor microenvironment through immune regulation presents potential therapeutic avenues for targeting EFTUD2 in cancer treatment. Nonetheless, more research is needed to fully understand its molecular mechanisms across different cancers and to explore its potential as a therapeutic target.

## 6. Role of EFTUD2 in non-neoplastic diseases

**Figure 6.**
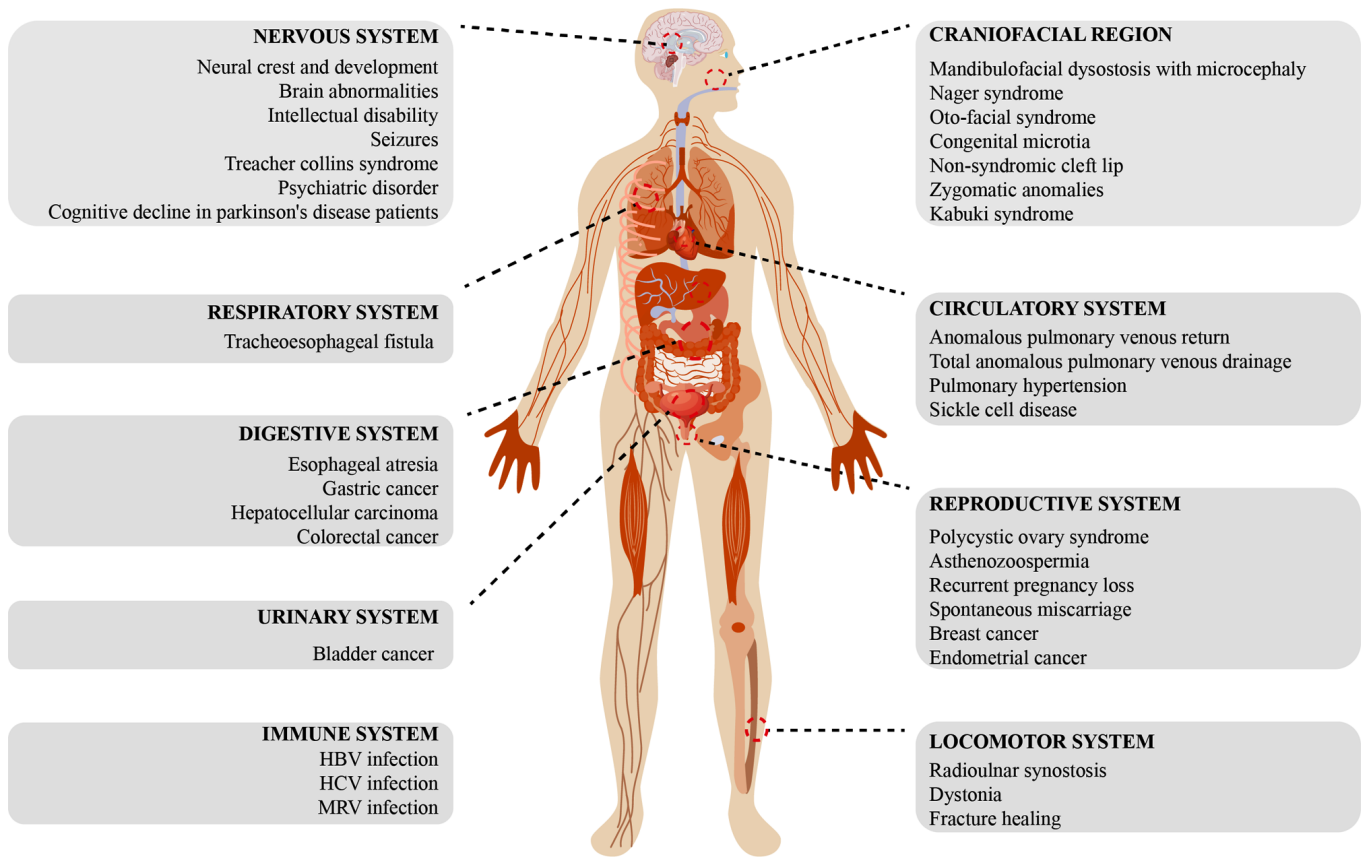
Distribution of EFTUD2-related diseases in the human body. The figure was created using Adobe Illustrator (version, 28.3; Adobe Systems, Inc.). EFTUD2, elongation factor Tu GTP binding domain containing 2.

Mutations in EFTUD2 can disrupt the normal function of the spliceosome, leading to RNA splicing errors, which manifest as various systemic defects and other associated diseases in the body (Fig. 6).

### MFD

MFD, commonly known as Treacher Collins syndrome, is a rare congenital disorder characterized by underdeveloped craniofacial bones (19,65). The etiology of MFD is multifactorial and recent studies emphasize that mutations in genes encoding major spliceosome core components are associated with various forms of this disease (66–68). MFD encompasses different subtypes, including MFDM and the MFDM Guion-Almeida type. The first description of MFDM emerged from a cohort of four unrelated Brazilian patients (69), marking an important step in recognizing the genetic foundation of this disorder. Since then, the identification of *EFTUD2* mutations through clinical genomic sequencing has become increasingly prevalent, underscoring the critical role of the gene in MFD pathology. Patients with *EFTUD2* mutations consistently exhibit significant mandibular hypoplasia characteristics (Table II) (35,40,70–73). Vincent *et al* (74) conducted genetic analysis of *EFTUD2* in 11 suspected cases of MFDM or MFD Guion-Almeida type. Among the cohort, four patients exhibited molecular abnormalities in *EFTUD2*, including missense mutations, nonsense mutations, duplications and deletions. All patients carrying *EFTUD2* mutations presented with microcephaly, hypoplasia of the zygomatic and mandibular bones, hearing loss, downslanting palpebral fissures and microtia (Table II). Bukowska-Olech *et al* (39) reported two female patients carrying novel heterozygous variants in *EFTUD2*, both presenting with mandibulofacial dysostosis of the Guion-Almeida type (Table II). Similarly, Luquetti *et al* (42) confirmed the presence of three novel variants in *EFTUD2* through Sanger sequencing, all of which were *de novo* variants. Two patients had missense mutations and one patient had a splice site mutation. All three patients exhibited significant mandibular hypoplasia (Table II). This pattern of symptoms reinforces the fact that *EFTUD2* mutations play a critical role in craniofacial development. *EFTUD2* is increasingly becoming a key gene that should be checked for mutations in the diagnosis or research on MFDM. Increasing types of *EFTUD2* mutations are being discovered, highlighting its significant role in craniofacial development and other developmental defect diseases. Especially in fetal screening, it provides a valuable reference to detect developmental defects.

### Nervous system

#### Intellectual disability and epilepsy

A case of a patient with mild intellectual disability was reported, in which the individual was identified to have a nonsense mutation in *EFTUD*2 (44). In a study involving 12 patients with congenital anomalies and/or intellectual disabilities and their trios, researchers identified two *EFTUD2* mutations through whole-exome sequencing (75). Matsuo *et al* (34), reported a case of a patient with epilepsy with early closure of the anterior fontanelle and patent ductus arteriosus at birth (Table II). The patient was found to have an *EFTUD2* mutation. An individual carrying an *EFTUD2* mutation was reported to suffer an initial seizure at 8 months old. Furthermore, an EEG indicated the presence of occasional spikes in the right frontal region (35). Another patient with generalized seizures and moderate intellectual disability was also found to have an *EFTUD2* splice site mutation (Table II) (44). Various signs indicate that *EFTUD2* mutations are probably an important factor in brain abnormalities, such as intellectual disabilities or epilepsy caused by developmental defects. However, no experimental studies have been reported so far and the specific mechanisms remain to be further explored.

### Psychiatric disorders

Park *et al* (19) constructed a quantitative model based on whole-genome sequencing information, which identified *EFTUD2* as an RNA binding protein (RBP) involved in diseases such as psychiatric disorders through RNA-RBP interaction profiling. Dysregulation of EFTUD2 plays a critical role in psychiatric disorders, such as attention deficit hyperactivity disorder and schizophrenia, by affecting target gene expression (76). The report indicated that variations in EFTUD2 and its downstream targets are associated with neurological diseases. However, the specific targets and mechanisms through which EFTUD2 is involved in intellectual disabilities have not been clarified and thus require further investigation.

### Cognitive impairment in patients with Parkinson's disease

Santiago and Potashkin (77) investigated 10 RNA biomarkers, including *EFTUD2* and compared their expression levels between patients with Parkinson's disease (PD) and healthy controls. The level of *EFTUD2* in cognitively normal PD patients (PD-CN) was markedly higher than in cognitively impaired PD patients (PD-MCI). The researchers performed a receiver operating characteristic (ROC) curve analysis on *EFTUD2*, which yielded an area under the curve (AUC) value of 0.64, suggesting that *EFTUD2* has some predictive value for PD-MCI. Current research is limited to clinical data analysis and whether EFTUD2 has a definitive impact on the progression of this disease requires further in-depth studies.

### Circulatory system

#### Anomalous pulmonary venous return

A male infant with TAPVD was reported to have an *EFTUD2* mutation (Table II) (36). Notably, the researchers observed that the infant's mother had mild facial asymmetry. Genomic sequencing revealed a heterozygous frameshift mutation in *EFTUD2* in both the mother and two of her infants (36). That study was the first to report a case of EFTUD2 haploinsufficiency presenting with TAPVD. The affected infants and their mother did not exhibit the classic phenotypic features of MFDM and the diagnosis was made solely through exome sequencing.

### Pulmonary hypertension

Wang *et al* (78) extracted data from 58 healthy controls and 135 patients with pulmonary hypertension from the Gene Expression Omnibus datasets, identifying *EFTUD2* as a differentially expressed hub gene. The authors used a hypoxic pulmonary hypertension rat model to confirm the significant upregulation of EFTUD2 in the pulmonary arteries of hypoxic pulmonary hypertension rats. The specific mechanisms of EFTUD2 in pulmonary arterial hypertension remain to be further elucidated.

### Sickle cell disease

In a bioinformatic analysis of Sickle Cell Disease, Liu *et al* (79) conducted a genome-wide association study (GWAS) on individuals with high and low hemoglobin (Hb) F levels. The GWAS data revealed single nucleotide polymorphisms (SNPs) in *EFTUD2* associated with high Hb F (79), hinting that *EFTUD2* is a potential new candidate locus. However, larger sample studies are required to confirm the role of EFTUD2 in γ-globin regulation.

### Digestive system

Researchers reported that esophageal atresia in patients was caused either by deletions in *EFTUD2* or novel heterozygous loss-of-function mutations in *EFTUD2*. These patients presented with severe micrognathia, upper airway obstruction, esophageal atresia, tracheoesophageal fistula and choanal atresia (Table II) (33,35,44,80). Wang *et al* (37) also reported a novel *de novo* frameshift deletion in the *EFTUD2* gene in a patient with EA/TEF. The patient's phenotype included EA/TEF, bilateral talipes equinovarus, hydrocele, atrial septal defect and renal pelvis dilatation (Table II). Khattar and *Suhrie* (38) also performed exome sequencing analysis on nine patients with EA/TEF and detected *EFTUD2* mutations in three patients. These studies indicate that mutations in *EFTUD2* can lead to severe gastrointestinal defects.

### Reproductive system

#### Polycystic ovary syndrome

In a screening study for characteristic genes of polycystic ovary syndrome (PCOS), Heidarzadehpilehrood *et al* (81) identified *EFTUD2* as a hub gene associated with PCOS. In addition, Hou *et al* (82) identified *EFTUD2* as a hub gene in the protein-protein interaction network of differential genes in PCOS. However, the specific function and mechanism of EFTUD2 in PCOS require further investigation.

### Recurrent pregnancy loss and spontaneous miscarriage

Yang *et al* (46) reported a study involving a non-consanguineous couple with a history of four consecutive clinical miscarriages at 10 weeks of gestation. A novel *de novo* nonsense mutation was identified in exon 12 of *EFTUD2* (Table II). The same mutation was found in 13.5% of sperm cells from the male partner, suggesting gonadal mosaicism. The researchers further confirmed the loss of *EFTUD2* gene function using a zebrafish model. Embryos carrying the *EFTUD2* mutation exhibited a significant reduction in the hindbrain neuronal marker paired box 2, as well as defects in the heart marker myosin light chain 7. Additionally, these embryos showed the common small head phenotype associated with *EFTUD2* mutations (46). It is noteworthy that the couple successfully conceived by selecting and implanting embryos without *EFTUD2* mutations and the pregnancy was confirmed through human chorionic gonadotropin testing and ultrasound (46).

### Asthenozoospermia

Li and Chen (83) identified *EFTUD2* as one of the genes with the highest connectivity degree in the differential gene network related to asthenozoospermia. They found that *EFTUD2* expression was downregulated in patients with asthenozoospermia. Further *in vivo* and *in vitro* studies are needed to confirm the potential mechanisms underlying these findings.

### Locomotor system

#### Limb deformities

In a sequencing study of 12 patients with Nager syndrome with limb anomalies, three were found to have *EFTUD2* mutations (84). Zarate *et al* (85) identified a heterozygous loss-of-function mutation in *EFTUD2* located on 17q21.31 in a patient with a rare syndrome characterized as the Guion-Almeida type. Compared with the previously mentioned three patients, this patient had a complete deletion of *EFTUD2* and exhibited a more pronounced phenotype of proximal radioulnar synostosis. This indicated the crucial role of EFTUD2 in skeletal development. However, the precise mechanisms of EFTUD2′s effect on radial bone development are not well understood and additional research is required to clarify EFTUD2′s role in limb anomalies.

### Dystonia

Zech *et al* (86) identified 14 pathogenic or potentially pathogenic CNVs, with *EFTUD2* being one of the most clinically relevant genes. The patients exhibited phenotypic features such as segmental dystonia with juvenile onset, facial dysmorphism, hearing impairment and autism spectrum disorder. A heterozygous single exon deletion mutation in *EFTUD2* (exon 11, 124bp) was also discovered, leading to MFDM and neurodevelopmental disorders with phenotypic variability.

### Infectious diseases

#### Anti-Hepatitis B virus (HBV) infection

**Figure 7.**
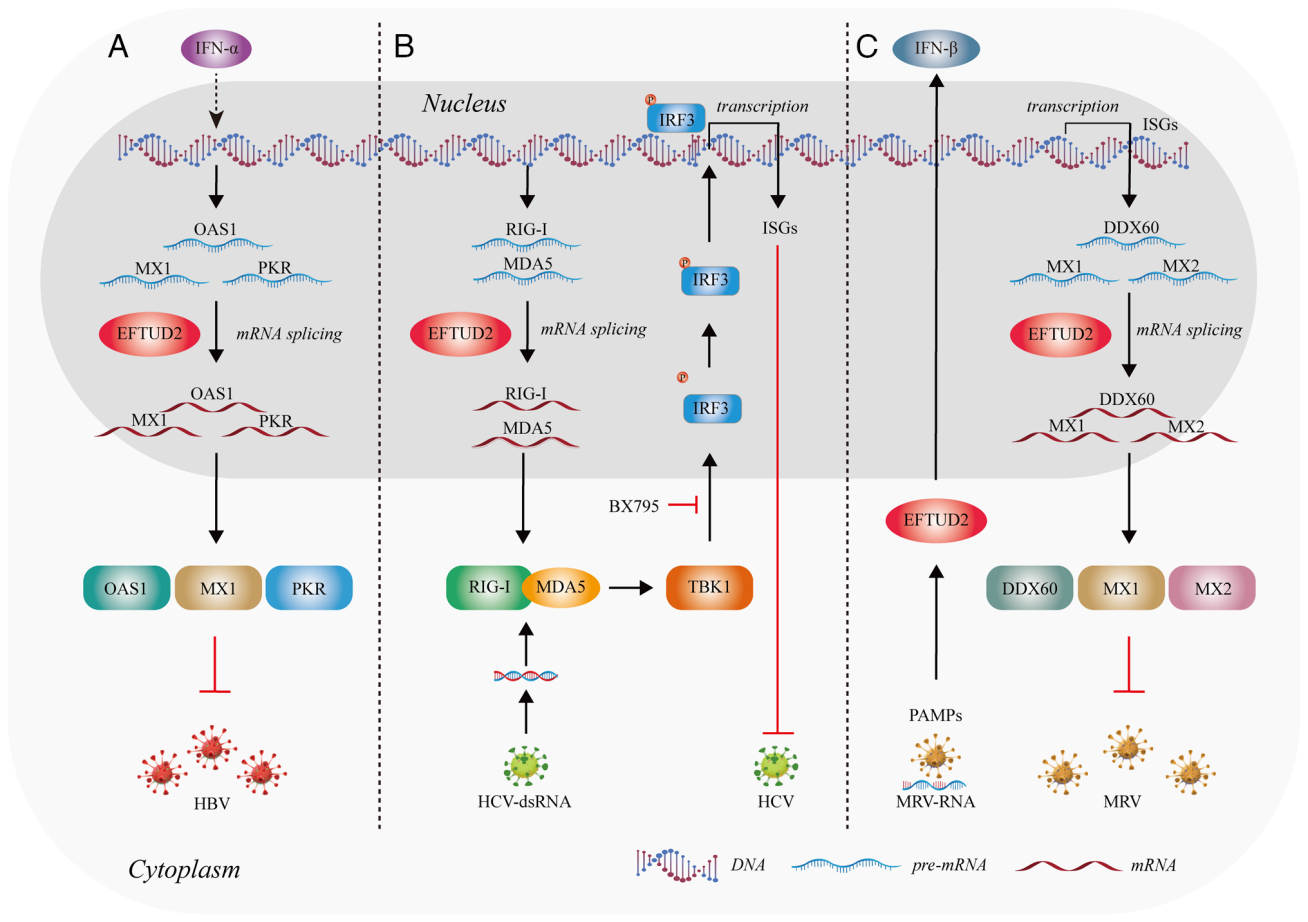
EFTUD2 is involved in antiviral processes by regulating splicing. (A) EFTUD2 participates in the anti-HBV response by controlling molecular splicing. (B) EFTUD2 regulates the splicing of antiviral proteins to inhibit HCV infection. (C) EFTUD2 participates in multiple antiviral mechanisms in response to MRV infection. The figure was created using Adobe Illustrator (version, 28.3; Adobe Systems, Inc.). EFTUD2, elongation factor Tu GTP binding domain containing 2; HBV, Hepatitis B virus; HCV, Hepatitis C virus; MRV, mammalian reovirus; IFN, interferon; OAS1, 2′-5′ oligoadenylate synthetase 1; MX1, MX dynamin-like GTPase 1; PKR, protein kinase R; IRF3, interferon regulatory factor 3; RIG-1, retinoic acid-inducible gene I; MDA5, melanoma differentiation-associated protein 5; TBK1, tank binding kinase 1; DDX60, DEAD-box helicase 60; PAMPs, pathogen-associated molecular patterns.

A study of 379 chronic HBV-infected individuals identified a SNP in *EFTUD2, EFTUD2*-rs3809756, which is associated with increased susceptibility to HBV infection (87). Individuals with the rs3809756-CC genotype have a higher risk of HBV infection compared with those with the rs3809756-AA genotype. Located in the promoter region of *EFTUD2*, the rs3809756 A>C polymorphism might reduce promoter activity (87). Following IFN-α treatment, *EFTUD2* knockout HepG2 cells showed a 5.43-fold increase in HBV DNA, a 2.80-fold increase in Hepatitis B surface antigen (HBsAg) and a 3.29-fold increase in Hepatitis B e-Antigen (HBeAg). The percentage of HBcAg-positive cells in HepG2.2.15 cells increased by 15% (88). Upon viral entry, HBV DNA is recognized through the combined action of retinoic acid-inducible gene I (RIG-I) and TLR3 (89,90). This pathogen recognition triggers downstream signaling pathways, leading to the expression of interferon-stimulated Genes (ISGs) (91,92). Research shows that EFTUD2 promotes the expression of key ISGs, such as MX dynamin-like GTPase 1 (MX1), 2′-5′-oligoadenylate synthetase and protein kinase R, by regulating mRNA splicing (88,93) to impede HBV infection (Fig. 7A). These proteins enhance the effects of type I IFNs and play a critical role in the host's innate immune defense. EFTUD2 might become a key target to exploring the therapeutic potential against HBV infection in future research.

### Anti-Hepatitis C Virus (HCV) and anti-Mammalian Reovirus (MRV) infection

Zhu *et al* (91) found that EFTUD2 inhibits HCV infection at 12 h post-infection and reaches a plateau at 24 h, indicating that EFTUD2 restricts HCV infection during the later stages of viral entry. EFTUD2 can also regulate the expression of RIG-I-like receptors RIG-I and melanoma differentiation-associated protein 5 (MDA5) through mRNA splicing. RIG-I and MDA5 can detect and recognize HCV RNA (94). This leads to the binding of RIG-I and MDA5 and activates the kinase TBK1, which then phosphorylates IRF3. Once phosphorylated, IRF3 forms dimers that translocate into the nucleus, subsequently inducing the expression of IFN genes and ISGs (95,96) (Fig. 7B). Therefore, EFTUD2 is essential for the activation of IRF3 and the expression of ISGs in TBK1-mediated anti-HCV responses downstream of RIG-I/MDA5 effectors.

EFTUD2 also restricted MRV replication in both single and multiple replication cycles (93), by regulating the recognition of viral pathogen-associated molecular patterns and the subsequent production of IFNs (93) (Fig. 7C). In addition, EFTUD2 elevates the basal mRNA levels of three ISGs:, MX1 and MX dynamin-like GTPase 2 to inhibit MRV infection (93) (Fig. 7C).

Overall, EFTUD2 exerts antiviral immune activity by regulating the expression of ISGs and cytokines through mRNA splicing. Exploring the innate immune mechanisms of EFTUD2 holds great potential for developing antiviral therapies.

In conclusion, EFTUD2 plays a crucial role in various non-neoplastic diseases, affecting craniofacial development, the nervous system, circulatory function, digestion, reproduction, the musculoskeletal system and immune responses to infections. Mutations in EFTUD2 are associated with a range of disorders, including craniofacial deformities, neurological impairments, circulatory dysfunction, reproductive issues and immune response regulation. Given its critical role in multiple pathological processes, EFTUD2 is a key gene for future clinical research and mechanistic exploration. Further studies on the molecular mechanisms through which EFTUD2 mutations lead to disease will help uncover potential therapeutic strategies and assess the feasibility of EFTUD2 as a treatment target.

## 7. Conclusion and perspectives

Collectively, EFTUD2 plays a vital role in fetal development and is frequently implicated in gene mutations linked to developmental defects. It is involved in the development and maintenance of nearly all bodily systems, including the craniofacial, nervous and respiratory systems. Thus, EFTUD2 shows potential as a target for gene therapy in the early treatment of fetal developmental defects.

In addition to its role in development, EFTUD2 is highly expressed in several cancers, including hepatocellular carcinoma, colorectal cancer, breast cancer and bladder cancer, in which it promotes tumor progression. However, research on EFTUD2 in cancer is still in its early stages. As a core component of the spliceosome, EFTUD2 holds significant potential for future research. Uncovering its molecular functions could provide new insights into the prevention and treatment of diseases such as cancer and developmental defects, particularly from the perspective of RNA splicing.

## Figures and Tables

**Table I.** EFTUD2 mutation sites and mutation types.

First author/s, year	EFTUD2 mutation site	Mutation type	Developmental defects in the patient	(Refs.)
Sarkar *et al*, 2015	c.933dupC (p.S312fs)	Frameshift	MFDM (Guion-Almeida type)	(32)
Smigiel *et al*, 2015	c.1435dup (p.Thr479AsnfsX2)	Frameshift	MFDM (Guion-Almeida type)	(33)
Matsuo *et al*, 2017	c.2698_2701del	Frameshift	MFDM (Guion-Almeida type)	(34)
Narumi-Kishimoto *et al*, 2020	c.2624dupT (p.Ile875fs)	Frameshift	MFDM (Guion-Almeida type)	(35)
McDermott *et al*, 2017	c.944delG (p.Ser315fs)	Frameshift	TAPVD, MFDM	(36)
Wang *et al*, 2021	c.2314del (p.Gln772ArgfsTer21)	Frameshift	EA/TEF	(37)
Khattar and Suhrie, 2023	c.969del	Frameshift	MFDM, EA/TEF	(38)
Smigiel *et al*, 2015	c.1859A>T (p.Lys620Met)	Missense	MFDM (Guion-Almeida type)	(33)
Lines *et al*, 2012	c.784C>T (p.Arg262Trp)	Missense	MFDM (Guion-Almeida type)	(40)
Bukowska-Olech *et al*, 2020	c.491A>G (p.Asp164Gly)	Missense	FD	(39)
Bukowska-Olech *et al*, 2020	c.779T>A (p.Ile260Asn)	Missense	FD	(39)
Lacour *et al*, 2019	c.2333C>A (p.Pro778His)	Missense	MFDM	(41)
Luquetti *et al*, 2013	c.2637G>A (p.Glu794Lys)	Missense	MFDM (*de novo* variant)	(42)
Luquetti *et al*, 2013	c.1458C>G (p.Gln401Glu)	Missense	MFDM (*de novo* variant)	(42)
Lacour *et al*, 2019	c.2466+1G>A (IVS24+1G>A)	Splice Site	MFDM	(41)
Luquetti *et al*, 2013	c.504-2G>T	Splice Site	MFDM	(42)
Voigt *et al*, 2013	c.994+1G>C	Splice Site	Oculo-Auriculo-Vertebral Spectrum (OAVS)	(44)
Voigt *et al*, 2013	c.2562-1G>C	Splice Site	MFDM, seizures	(44)
Khattar and Suhrie, 2023	c.1058+1G>A	Splice Site	EA/TEF	(38)
Kim *et al*, 2020	c.271+1G>A	Splice Site	MFDM	(43)
Voigt *et al*, 2013	c.351-1G>A (p.Asp117Glufs*8)	Splice Site	Oto-facial syndrome	(44)
Voigt *et al*, 2013	c.594T>G (p.Tyr198*)	Nonsense	MFDM, intellectual disability	(44)
Rengasamy Venugopalan *et al*, 2017	c.259C>T (p.Gln87*)	Nonsense	MFDM (*de novo* variant)	(45)
Sarkar *et al*, 2015	c.1732C>T (p.R578X)	Nonsense	MFDM	(32)
Yang *et al*, 2022	c.1012G>T (p.E338*)	Nonsense	MFDM, gonadal mosaicism	(46)

EFTUD2, elongation factor Tu GTP binding domain containing 2; MFDM, mandibulofacial dysostosis with microcephaly; TAPVD, total anomalous pulmonary venous drainage; EA/TEF, esophageal atresia/tracheoesophageal fistula; FD, facial dysostosis; OAVS, oculo-auriculo-vertebral spectrum; */X, represents stop codon.

**Table II.** Clinical symptoms of patients with EFTUD2 mutations.

First author/s, year	EFTUD2 mutation site	Clinical symptoms of patients	(Refs.)
Sarkar *et al*, 2015	c.933dupC (p.S312fs)	Malar hypoplasia, mandibular hypoplasia, microcephaly, abnormal external ears, hearing loss, developmental delay	(32)
Smigiel *et al*, 2015	c.1435dup (p.Thr479AsnfsX2)	Cleft palate, microcephaly, facial asymmetry, palpebral fissures downslanting, absent/sparse lateral lower eyelashes, hyperplastic supraorbital ridges, malar hypoplasia, broad base of nose, microtia, low set ears, preauricular tags, dysplastic ears, micrognathia, proximally placed thumbs, brachydactyly, esophageal atresia and tracheoesophageal fistula, hearing loss, feeding problems, gastrostomy, tracheostomy, psychomotor delay, speech delay, somatic delay and microsomia	(33)
Matsuo *et al*, 2017	c.2698_2701del	Microcephaly, malar hypoplasia, mandibular hypoplasia, deafness, epilepsy and developmental delay	(34)
Narumi-Kishimoto *et al*, 2020	c.2624dupT (p.Ile875fs)	Micrognathia, malar hypoplasia, microcephaly, abnormality of the pinna, ossicular abnormalities, hearing impairment, cleft palate, seizures, esophageal atresia, scoliosis/kyphosis, growth failure, intellectual disability and choanal atresia	(35)
McDermott *et al*, 2017	c.944delG (p.Ser315fs)	Total anomalous pulmonary venous drainage, tracheoesophageal fistula, facial palsy or asymmetry and esophageal Atresia	(36)
Wang *et al*, 2021	c.2314del (p.Gln772ArgfsTer21)	EA/TEF, atrial septal defect, bilateral clubfoot, hydrocele and renal pyelectasis	(37)
Khattar and Suhrie, 2023	c.969del	EA/TEF, micrognathia, microcephaly, accessory ear tags, mitral valve stenosis, cleft palate, left ear microtia, right preauricular tag, exotropia, amblyopia, astigmatism and mild left kidney pelviectasis	(38)
Smigiel *et al*, 2015	c.1859A>T (p.Lys620Met)	Microcephaly, facial asymmetry, palpebral fissures downslanting, lacrimal duct anomalies, hypertelorism, malar hypoplasia, broad base of nose, preauricular tags, dysplastic ears, micrognathia, proximally placed thumbs, camptodactyly, choanal atresia, hearing loss, psychomotor delay, speech delay, somatic delay and microsomia	(33)
Lines *et al*, 2012	c.784C>T (p.Arg262Trp)	Hyperplastic supraorbital ridges, hypertelorism, malar hypoplasia, broad base of nose, low set ears, dysplastic ears, micrognathia, proximally placed thumbs, choanal atresia, hearing loss, ophthalmology problems, astigmatism, myopia, ptosis, strabismus feeding problems, gastrostomy, tracheostomy, psychomotor delay, speech delay	(40)
Bukowska-Olech *et al*, 2020	c.491A>G (p.Asp164Gly)	Trigonocephaly, upturned nose and preaxial polydactyly	(39)
Bukowska-Olech *et al*, 2020	c.779T>A (p.Ile260Asn)	Intellectual impairment, delayed psychomotor development, delayed speech development, epilepsy, microcephaly, trigonocephaly, midface hypoplasia, malar hypoplasia, micrognathia, buccal tags, preauricular tag, preauricular pit, low-set ears, dysplastic ears, conductive hearing loss, upslanting palpebral fissures, downslanting palpebral fissures, short nose and atrial septal defect	(39)
Lacour *et al*, 2019	c.2333C>A (p.Pro778His)	Hemifacial microsomia, cleft lip and palate, mild microcephaly, dysplastic ears and hearing loss	(41)
Luquetti *et al*, 2013	c.2637G>A (p.Glu794Lys)	Facial asymmetry, choanal atresia, epibulbar dermoid, cleft of left zygomatic arch, bilateral microtia, preauricular skin tags, small external auditory canal, hearing loss, incompletely formed lateral semicircular canal and dilated vestibule, mandibular hypoplasia, malar hypoplasia, micrognathia, cleft palate, thumb abnormalities, developmental delay	(42)
Luquetti *et al*, 2013	c.1458C>G (p.Gln401Glu)	Microcephaly, facial asymmetry, choanal atresia, cleft of zygomatic arch, bilateral microtia, preauricular skin tags, atretic external auditory canal, hearing loss, dysplastic ossicles, mandibular hypoplasia, malar hypoplasia, micrognathia, developmental delay, seizures, malformed ossicles, mandibular asymmetry, thumb abnormalities, cervical spine abnormalities, developmental delay, seizures	(42)
Lacour *et al*, 2019	c.2466+1G>A (IVS24+1G>A)	Left hemifacial microsomia, left ear canal atresia with third-degree microtia, presence of a lobule in anomalous position, the upper half of the partial pinna located posteriorly, metopic craniosynostosis with trigonocephaly, VSD, PFO and mild diffuse atrophy of the brain	(41)
Luquetti *et al*, 2013	c.504-2G>T	Microcephaly, facial asymmetry, choanal atresia, cleft of zygomatic arch, bilateral microtia, preauricular skin tags, atretic external auditory canal, hearing loss, dysplastic ossicles, mandibular hypoplasia, malar hypoplasia, micrognathia, developmental delay, seizures	(42)
Voigt *et al*, 2013	c.994+1G>C	Polyhydramnios, facial asymmetry, upslanting palpebral fissures, microtia/with squared earlobe, a-/hypoplasia of external ear canal, hearing loss, cleft palate, reduced mouth opening, micrognathia, malformations tracheostomy, esophageal atresia, CHD, scoliosis left of zygomatic bone, clinodactyly V	(44)
Voigt *et al*, 2013	c.2562-1G>C	Epilepsy, hyperplastic supraorbital ridges, Frontal bossing, Microtia/with squared earlobe, preauricular tag, preauricular pit, a-/hypoplasia of external ear canal, hearing loss, nasal speech, reduced mouth opening, micrognathia	(44)
Khattar and Suhrie, 2023	c.1058+1G>A	EA/TEF, microcephaly, micrognathia, hyperopia and astigmatism, microtia	(38)
Kim *et al*, 2020	c.271+1G>A	Abnormal echogenicity in the pulmonary artery area, tricuspid valve insufficiency,	(43)
Voigt *et al*, 2013	c.351-1G>A (p.Asp117Glufs*8)	Polyhydramnios, facial asymmetry, upslanting palpebral fissures, microtia/with squared earlobe, A-/hypoplasia of external ear canal, hearing loss, cleft of zygomatic bone, choanal atresia, small middle ear cavity	(44)
Voigt *et al*, 2013	c.594T>G (p.Tyr198*)	Polyhydramnios, upslanting palpebral fissures, microtia/with squared earlobe, A-/hypoplasia of external ear canal, cleft palate, micrognathia, esophageal atresia, inner/middle ear malformations	(44)
Rengasamy Venugopalan *et al*, 2017	c.259C>T (p.Gln87*)	Gross facial asymmetry, micrognathia, airway obstruction, choanal atresia, left ear microtia, bilateral absence of ear canals and conductive hearing loss, speech articulation problems and microcephaly	(45)
Sarkar *et al*, 2015	c.1732C>T (p.R578X)	Malar hypoplasia, mandibular hypoplasia, microcephaly, abnormal external ears, hearing loss, developmental delay, auditory canal defects, inner ear abnormalities	(32)
Yang *et al*, 2022	c.1012G>T (p.E338*)	Recurrent pregnancy loss	(46)

EFTUD2, elongation factor Tu GTP binding domain containing 2; EA/TEF, esophageal atresia/tracheoesophageal fistula; VSD, ventricular septal defect; PFO, patent foramen ovale.

**Table III.** Research progress for EFTUD2 in tumors.

First author/s, year	Tumor type	EFTUD2 expression	Mechanisms	Effects	Function	(Refs.)
Tu *et al*, 2020; Lv *et al*, 2022; Zhou, *et al*, 2022; Zhou *et al*, 2021; Chi *et al*, 2020	Hepatocellular carcinoma	UP	Regulation of methylation; reduction of YTHDF3 ubiquitination; promotion of the STAT3 pathway	Enhanced lactate production; Promotion of cell cycle progression; Inhibition of apoptosis; Promotion of EMT; Immune infiltration; Reduced prognosis	Biomarker	(47–49,51,97)
Beyer *et al*, 2023	Endometrial Cancer	UP	-	Reduced prognosis	Biomarker	(64)
Sato *et al*, 2015	Breast Cancer	UP	Binding with SNW1	Inhibition of apoptosis	Biomarker	(61)
Chen *et al*, 2021; Zhang *et al*, 2023	Bladder Cancer	UP	-	Reduced prognosis	Diagnostic Biomarker	(63,98)
Lv *et al*, 2019	Colorectal Cancer	UP	Activation of TLR4 signaling and NF-κB; TLR4/MD-2/MyD88 pathway	Reduced prognosis; The occurrence of colitis-associated cancer	Biomarker	(52)

UP, upregulated; EFTUD2, elongation factor Tu GTP binding domain containing 2; YTHDF3, YTH domain family protein 3; STAT3, signal transducer and activator of transcription 3; SNW1, SNW domain containing 1; TLR4, Toll-like receptor 4; NF-κB, nuclear factor kappa B; MD-2, myeloid differentiation protein-2 MyD88, myeloid differentiation primary response 88.

## Data Availability

Not applicable.

## Abbreviations

EFTUD2: elongation factor Tu GTP binding domain containing 2
GTP: guanosine-5′-triphosphate
snRNP: small nuclear ribonucleoproteins
MFD: mandibulofacial dysostosis
MFDM: mandibulofacial dysostosis with microcephaly
EJC: exon junction complex
MX: myxovirus resistance
MyD88: myeloid differentiation primary response 88
HSP90: heat shock protein 90β
SAMHD1: sterile alpha motif and histidine-aspartate domain-containing protein 1
OAVS: oculo-auriculo-vertebral spectrum
PFKL: phosphofructokinase L
YTHDF3: YTH domain family protein 3
BRR2: small nuclear ribonucleoprotein U200
